# A single-cell chromatin accessibility dataset of human primed and naïve pluripotent stem cell-derived teratoma

**DOI:** 10.1038/s41597-024-03558-9

**Published:** 2024-07-02

**Authors:** Jinxiu Li, Lixin Fu, Yunpan Li, Wei Sun, Yao Yi, Wenqi Jia, Haiwei Li, Hao Liu, Pengcheng Guo, Yang Wang, Yue Shen, Xiuqing Zhang, Yuan Lv, Baoming Qin, Wenjuan Li, Chuanyu Liu, Longqi Liu, Md. Abdul Mazid, Yiwei Lai, Miguel A. Esteban, Yu Jiang, Liang Wu

**Affiliations:** 1 College of Life Sciences, University of Chinese Academy of Sciences, Beijing, 100049 China; 2 BGI Research, Shenzhen, 518083 China; 3 BGI Research, Hangzhou, 310030 China; 4 Laboratory of Integrative Biology, Guangzhou Institutes of Biomedicine and Health, Chinese Academy of Sciences, Guangzhou, 510530 China; 5 College of Life Sciences, Nankai University, Tianjin, 300071 China; 6 MRC Metabolic Diseases Unit, Wellcome Trust-Medical Research Council Institute of Metabolic Science, University of Cambridge, Cambridge, CB2 0QQ UK; 7 Joint School of Life Sciences, Guangzhou Institutes of Biomedicine and Health and Guangzhou Medical University, Guangzhou, Guangdong 510530 China; 8 BGI Research, Changzhou, 213299 China; 9 3DCStar lab, BGI, Shenzhen, 518083 China; 10 State Key Laboratory for Diagnosis and Treatment of Severe Zoonotic Infectious Diseases, Key Laboratory for Zoonosis Research of the Ministry of Education, Institute of Zoonosis, and College of Veterinary Medicine, Jilin University, Changchun, 130062 China

**Keywords:** Cell lineage, Next-generation sequencing

## Abstract

Teratoma, due to its remarkable ability to differentiate into multiple cell lineages, is a valuable model for studying human embryonic development. The similarity of the gene expression and chromatin accessibility patterns in these cells to those observed *in vivo* further underscores its potential as a research tool. Notably, teratomas derived from human naïve (pre-implantation epiblast-like) pluripotent stem cells (PSCs) have larger embryonic cell diversity and contain extraembryonic lineages, making them more suitable to study developmental processes. However, the cell type-specific epigenetic profiles of naïve PSC teratomas have not been yet characterized. Using single-cell assay for transposase-accessible chromatin sequencing (scATAC-seq), we analyzed 66,384 cell profiles from five teratomas derived from human naïve PSCs and their post-implantation epiblast-like (primed) counterparts. We observed 17 distinct cell types from both embryonic and extraembryonic lineages, resembling the corresponding cell types in human fetal tissues. Additionally, we identified key transcription factors specific to different cell types. Our dataset provides a resource for investigating gene regulatory programs in a relevant model of human embryonic development.

## Background & Summary

Studying the mechanisms of early cell fate determination is crucial not only for understanding normal human embryo development but also for addressing the effect of developmental disorders^1–3^. However, ethical concerns and the scarcity of human embryos compel the reliance on animal models, such as mice, to gain insights into these fundamental processes^4–7^. While the models have provided valuable information, the highly species-specific nature of development highlights the limitations of extrapolating findings to humans. For instance, human gastrulation unfolds in a planar embryonic disc, contrasting with the formation of an egg cylinder in mice^8^. Furthermore, distal visceral endoderm (DVE), which play significant roles in anterior-posterior axis formation in mice gastrulation, is absent in primates^9^. Consequently, exploring the unique aspects of human development requires suitable models.

Teratomas, generated by the implantation of pluripotent stem cells (PSCs) into the murine dermic layer, have emerged as an encouraging *in vivo* model for recapitulating relevant aspects of human developmental processes^10^. This offers significant practical advantages in terms of ethical concerns and ease of implementation^11,12^. Teratomas derived from human primed PSCs generate tissue-like structures representing all three germ layers^13^. Importantly, those derived from naïve PSCs exhibit an expanded capacity to generate multi-lineage cell types across not only embryonic but also extraembryonic cell lineages compared to primed PSCs^11,14,15^. Single-cell RNA sequencing (scRNA-seq) has confirmed the capacity of producing diverse cell types across various developmental lineages in primed and naïve PSC teratomas^11,14,15^. Chromatin accessibility, the degree to which the chromatin structure is open and accessible to transcription factors, RNA polymerases and other regulatory proteins, plays a pivotal role in regulating gene expression and orchestrating cell fate transitions^16,17^. Proper reconfiguration of the chromatin landscape ensures a cell-state-specific gene expression profile, thereby facilitating smooth cell state transitions^18–20^. While single-cell assay for transposase-accessible chromatin sequencing (scATAC-seq) has been applied to teratomas derived from primed PSCs to study open chromatin, a comprehensive study comparing primed and naïve PSCs is still lacking.

In this study, we used scATAC-seq to generate a dataset encompassing 66,384 cell profiles. These profiles were obtained from five teratomas, of which three derived from human naïve PSCs, and the remaining two from primed PSCs. We identified 17 distinct cell types, covering both embryonic and extraembryonic lineages, resembling corresponding cell types in human fetal tissues. Further analysis pinpointed key transcription factors enriched in cell type-specific accessible regions of chromatin, such as SPI1 (transcription factor PU.1) in myeloid cells and TFAP2C (transcription factor AP-2γ) in extraembryonic lineages. Our dataset is valuable for dissecting the cis-regulatory logic of embryonic cell fate specification during multilineage differentiation, enhancing our understanding of normal human embryonic development, and offering insights potentially useful for disease prevention and producing functional cells for therapy.

## Methods

### Animal study and ethics statement

All mouse experiments were approved by and conducted in accordance with the guidelines set by the corresponding ethics committees of BGI (license number: BGI-IRB A23019) and the Guangzhou Institutes of Biomedicine and Health (license numbers: IACUC2021002 and GIBH-LMEC2023-004-01 (AL)).

### Human primed PSC culture

Human H9 embryonic stem cells (ESCs) were obtained from WiCell Research Institute. Primed human ESCs were routinely cultured in mTeSR^TM^1 medium (Stemcell Technologies, 85850) on plates coated with extracellular matrix (Geltrex (ThermoFisher, A1413302) or Matrigel (Corning, 354277)), and the medium was refreshed every day. In general, primed PSCs were passaged every 4 days. For passaging, cells were washed once with DPBS (Hyclone, SH30028.02) and treated with 0.5 mM EDTA (Invitrogen, 15575020) for 5 minutes. EDTA was then removed, and the cells were passaged as small clumps using a Pasteur pipette (Greiner bio-one, 612361).

### Generation of human naïve PSCs

Feeder cells derived from ICR mouse embryonic fibroblasts (mitomycin-C treated) were maintained around 24 hours in DMEM (Corning, 10-013-CMR) supplemented with 10% FBS (Natocor, SFBE) before using them for generating 4CL naïve PSCs. To generate 4CL naïve PSCs, primed human H9 ESCs were washed with DPBS, then cells were dissociated into single cells with TrypLE (Gibco, 12604-021). The resulting single cells were plated at a density of 1,000–1,500 cells/cm^2^ on feeders in mTeSR^TM^1 medium supplemented with 10 µM Y-27632 (Axon, 1683) and 24 hours later the medium was switched to 4CL medium composed of 1:1 mix of Neurobasal medium (Gibco, 21103049) and Advanced DMEM/F12 (Gibco, 12634028) supplemented with N2 (Gibco, 17502048) and B27 (Gibco, 17504044), sodium pyruvate (Corning, 25000CL), non-essential amino acids (Corning, 25025CL), GlutaMAX (Gibco, 35050061), penicillin–streptomycin (HyClone, SV30010), 10 nM DZNep (Selleck, S7120), 5 nM TSA (Vetec, V900931), 1 µM PD0325901 (Axon, 1408), 5 µM IWR-1 (Sigma, I0161), 20 ng ml^–1^ human LIF (Peprotech, 300-05), 20 ng ml^–1^ activin A (Peprotech, 120-14E), 50 µg ml^–1^ l-ascorbic acid (Sigma, A8960) and 0.2% (v/v) Geltrex or Matrigel. 4CL medium was refreshed every day, and cells were passaged as single cells (1:5 to 1:8) every 3–4 days; optionally, 5 µM Y-27632 was added in the medium for the first 24 hours after passaging.

### Teratoma formation

For teratoma formation, a 200 µl pre-chilled 1:1 mixture of DMEM/F12 and Matrigel was used to suspend 1 million cells. Male NOD-SCID IL2Rg^−/−^ mice aged 6–8 weeks were subcutaneously injected with this cell suspension. Teratomas were collected approximately 9 weeks post-injection and were directly processed to prepare cell suspensions for scATAC-seq.

### scATAC-seq library preparation and sequencing

ScATAC-seq libraries were prepared using the DNBelab C Series Single-Cell ATAC Library Prep Set^21^ (MGI, 1000021878). The process involved converting transposed single-nucleus suspensions into barcoded scATAC-seq libraries through several steps: droplet encapsulation, pre-amplification, emulsion breakage, beads collection, DNA amplification, and purification. The DNA concentration of each library was measured using a Qubit ssDNA Assay kit and sequenced on a DIPSEQ T1 sequencer at the China National GeneBank (CNGB).

### scATAC-seq raw data processing

ScATAC-seq datasets were processed using the DNBelab C Series scATAC-seq standard analysis pipeline, which included fastq debarcoding, read trimming, alignment, bead filtration, and bead deconvolution. In brief, raw reads were filtered and demultiplexed using PISA (v0.12)^22^, allowing for 2 mismatches in the barcode. The retained reads were then aligned to the human genome using BWA-MEM (v0.7.15)^23^ with default parameters. Reads with a mapping quality below 10 and PCR duplicates were removed from each library using Picard (v1.84, https://github.com/broadinstitute/picard). The final fragments for each library were utilized for downstream analysis using the ArchR package (v1.0.1)^24^.

### Dimensionality reduction, clustering and cell type annotation

Analyses were conducted using the ArchR package^24^. Arrow file of each library was created using ‘createArrowFiles’ function with ‘filterFrags’ set 50 by reading in accessible read fragments for each library. The gene activity score matrix, a predicted gene expression matrix based on the accessibility of each gene loci, was calculated using ‘addGeneScoreMatrix’ function with default parameter. Cell with fewer than 1,200 fragments or a TSS (transcription start site) enrichment lower than 7 were excluded. Potential doublets were detected and removed using the ‘filterDoublets’ function with default parameters. Uniform Manifold Approximation and Projection (UMAP) embedding was performed based on Iterative Latent Semantic Indexing (LSI) dimensionality, which generated using the top 25,000 variable genome tiles. Cell clusters were identified using the Shared Nearest Neighbor (SNN) graph based on LSI dimensions. Cell types were annotated by manually inspecting the ‘gene activity score’ and accessible regions of canonical marker genes.

### Correlation analysis with *in vivo* data

To validate each annotated cell type from teratomas, we performed a correlation analysis between the cell types in the teratomas and *in viv*o human fetal tissues. The Pearson correlation was computed between the average gene activity score of each cell type and the average expression of each broad cell type from published fetal organogenesis datasets (scRNA-seq data, *in vivo* 72–129 days)^1^. The union of all marker genes for teratoma cell types and fetal organogenesis cell types was used as features for calculating the correlation.

### Peak calling and DAR motif enrichment

For peak calling, the ‘addGroupCoverages’ function of ArchR package^24^ was first used to create pseudo-bulk replicates for each cell type, then peaks were defined using the ‘addReproduciblePeakSet’ function with default parameters. For motif enrichment analysis, cell-type differential peak analysis was performed using the ‘getMarkerFeatures’ function (FDR < 0.05 & Log2 FC > 1). Enriched motifs for each cell type were identified using the ‘peakAnnoEnrichment’ function. For heatmap charting, motif enrichment (-log_10_ (*P* value)) greater than 5 were preserved.

### Transcription factor (TF) footprinting analysis

Relevant motif positions were extracted using the ‘getPositions’ function of ArchR package^24^ with default parameters. ‘addGroupCoverages’ and ‘getFootprints’ were then used to obtain cell type-specific TF footprints. Finally, these TF footprints were visualized using the ‘plotFootprints’ function.

## Data Records

All raw fastq data of scATAC-seq generated by this study have been deposited to CNGB Nucleotide Sequence Archive (CNSA)^25^ of CNGB DataBase (CNGBdb)^26^ (accession number: CNP0004965)^27^ and Sequence Read Archive (SRA) of NCBI (accession number: SRP504852)^28^. Additionally, fragments of each library and cell annotations were uploaded to Figshare^29^ (10.6084/m9.figshare.24547609.v2). The cell-type peak matrices and motif enrichment in these marker peaks were also uploaded to Figshare^29^ (10.6084/m9.figshare.24547609.v2).

## Technical Validation

**Fig. 1:**
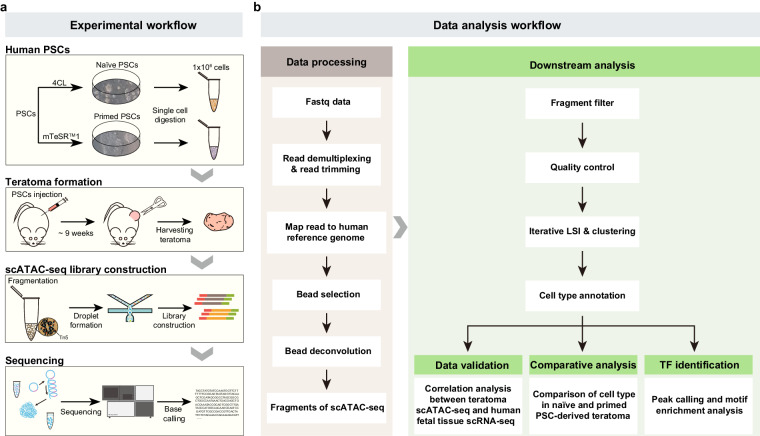
Schematic depicting the key experiments and data analysis. (**a**) Key experimental workflow. PSCs: pluripotent stem cells. (**b**) Main steps of data processing and analysis.

In this study, we conducted a teratoma formation assay with human PSCs in different pluripotent states (naïve PSCs cultured in 4CL medium^15^ and primed PSCs cultured in mTeSR^TM^1 medium). Teratoma were formed with high frequency from both naïve (100%) and primed (100%) PSCs. Specifically, three teratomas were derived from naïve PSCs and two arose from primed PSCs. These teratomas were collected at approximately nine weeks and subjected to single-cell digestion. Subsequently, their chromatin accessible regions were detected using the DNBelab C Series Single-Cell ATAC library Prep set^21^ (Fig. 1a). The resulting fastq data was processed through a well-established standard pipeline (Fig. 1b).

**Fig. 2:**
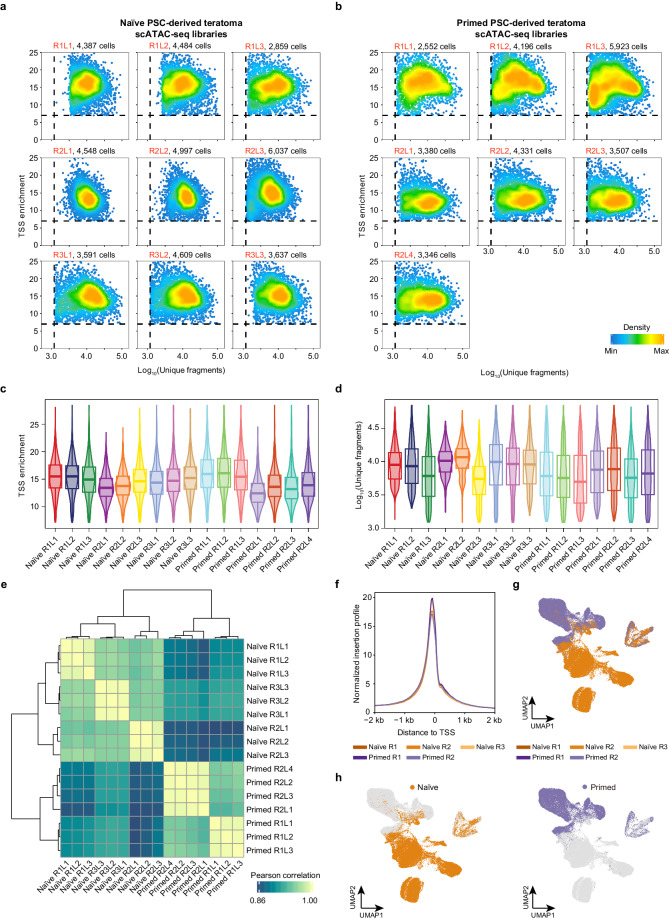
Quality control of scATAC-seq dataset. (**a,** **b**). Scatter heatmap showing the distributions of the TSS enrichment scores and unique fragments of individual libraries, generated from naïve PSC-derived teratomas (**a**), and primed PSC-derived teratomas (**b**). R: biological replicate; L: library. (**c,** **d**). Violin plot illustrating the distribution of unique fragments (**c**) and TSS enrichment score (**d**) for each library. (**e**) Heatmap showing the Pearson correlation among the libraries in this study based on the gene activity score. (**f**) Line plot showing the enrichment of scATAC-seq signals around TSS. Biological replicates of teratomas are highlighted by different colors. (**g**) UMAP showing the five corresponding biological replicates of naïve and primed PSC-derived teratomas. (**h**) UMAP highlighting the cells of naïve (left panel) and primed (right panel) PSC-derived teratomas.

After a stringent quality control, a total of 66,384 high-quality cells were obtained (Fig. 2a,b). Each library contained a median of 7,764 distinct DNA fragments and showed a median TSS enrichment of 14.487 (Fig. 2c,d, Table 1). The analysis yielded 27,235 cells from naïve PSC-derived teratomas and 39,149 cells from primed PSC-derived teratomas. To evaluate the reproducibility between replicates, Pearson correlation coefficient was calculated based on the average ‘gene activity score’, which revealed a high consistency among both technical and biological replicates (Fig. 2e). Furthermore, the biological replicates displayed strong and similar enrichment signals around the TSS region (Fig. 2f). Additionally, UMAP representation revealed that the biological replicates showed a uniform pattern of chromatin accessibility (Fig. 2g), indicating consistent developmental processes of the same ESC state. Although the distribution between naïve and primed PSC-derived teratomas differed, this suggested differentiation heterogeneity of distinct ESC states (Fig. 2g,h), in accordance with our previous observations using scRNA-seq^15^.

**Table 1:** Statistical measures for the scATAC-seq profiles of individual libraries. R: biological replicate; L: library.

Sample ID	Cell number	Median fragment	TSS enrichment	Fragments in peaks	Fragment in peaks (%)	Peak number
Naïve R1L1	4,387	9,091	15.54	11,515	65.54%	44,402
Naïve R1L2	4,484	8,576	15.38	10,652	64.28%	50,897
Naïve R1L3	2,859	6,099	14.89	7,161	62.85%	68,822
Naïve R2L1	4,548	9,993	13.42	11,248	58.95%	58,683
Naïve R2L2	4,997	11,551	13.77	13,093	58.68%	54,557
Naïve R2L3	6,037	5,460	14.70	6,384	60.63%	76,324
Naïve R3L1	3,591	10,053	14.39	11,930	60.73%	59,171
Naïve R3L2	4,609	9,027	14.74	10,839	62.52%	55,745
Naïve R3L3	3,637	8,728	15.02	10,729	63.02%	60,970
Primed R1L1	2,552	6,110	15.85	7,069	60.65%	61,537
Primed R1L2	4,196	5,507	16.26	6,410	60.68%	58,753
Primed R1L3	5,923	5,120	15.49	5,604	59.68%	98,031
Primed R2L1	3,380	7,457	12.50	9,793	67.45%	61,334
Primed R2L2	4,331	7,943	13.53	10,541	68.14%	66,526
Primed R2L3	3,507	5,655	13.20	7,542	67.95%	53,815
Primed R2L4	3,346	6,663	13.93	9,024	68.89%	83,840

**Fig. 3:**
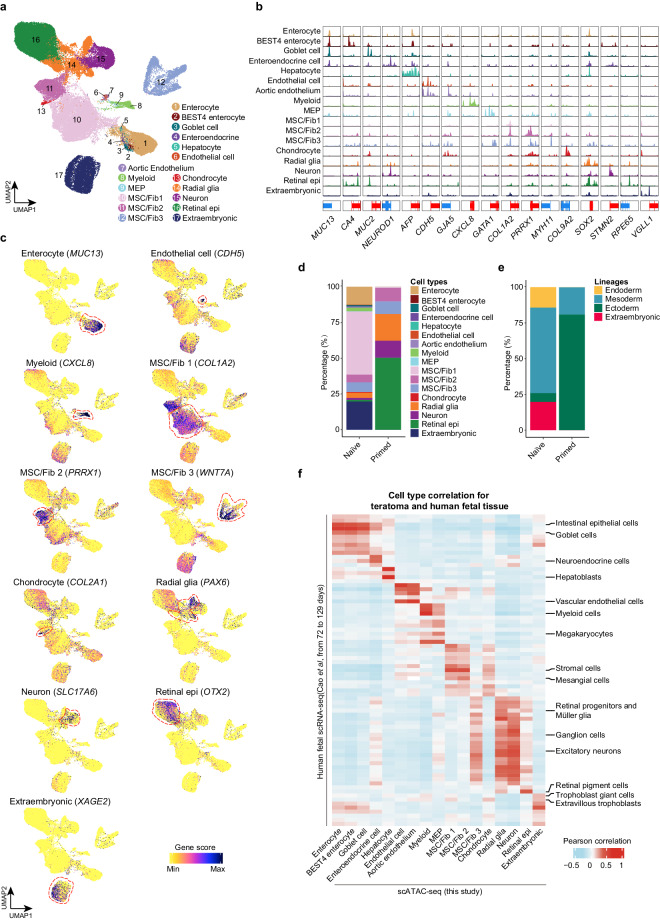
Chromatin landscape of teratoma and association with *in vivo* human fetal tissues. (**a**) UMAP showing the identified 17 cell types in scATAC-seq of naïve and primed PSC-derived teratomas. MEP: megakaryocyte-erythroid progenitor, Retinal epi: retinal epithelial cell, MSC/Fib: mesenchymal stem cell/fibroblast. (**b**) Aggregated chromatin accessibility of each cell type at representative marker gene loci in scATAC-seq data (±8 kb around TSS). (**c**) UMAP showing the gene activity score of cell type-specific gene. (**d**) Bar plot showing the percentage of cell types in naïve and primed PSC-derived teratomas. (**e**) Bar plot showing the percentage of lineages in naïve and primed PSC-derived teratomas. (**f**) Heatmap showing the Pearson correlation coefficients between cell type averaged ‘gene activity score’ of scATAC-seq for teratomas and cell type averaged gene expression of scRNA-seq for human fetal tissues^1^.

Next, we uncovered cell type annotations from the open chromatin information. Based on the chromatin accessibility of known marker genes, we identified 17 putative cell types in the teratomas (Fig. 3a–c). These cell types encompassed three germ layer-derived (embryonic) and extraembryonic lineages. Specifically, within the endoderm lineage, we identified enterocyte (*MUC13*^+^)^30^, BEST4 enterocyte (*MUC13*^+^, *CA4*^+^)^31^, goblet cell (*MUC13*^+^, *MUC2*^+^)^31^, enteroendocrine (*MUC13*^+^, *NEUROD1*^+^)^31^ and hepatocyte (*AFP*^+^)^32^. In the mesoderm lineage, we found endothelial cell (*CDH5*^+^)^33^, aortic endothelium (*CDH5*^+^, *GJA5*^+^)^34^, myeloid cell (*CXCL8*^+^)^35^, megakaryocyte-erythroid progenitor (MEP, *GATA1*^+^)^36^, chondrocyte (*COL2A1*^+^, *COL9A2*^+^)^37^ and three types of mesenchymal stem cell (MSC)/fibroblast (Fib): *COL1A2*^+^ for MSC/Fib 1, *PRRX1*^+^ for MSC/Fib 2, *MYH11*^+^ and *WNT7A*^+^ for MSC/Fib 3^38–40^. Within the ectoderm lineage, we identified radial glia (*SOX2*^+^, *PAX6*^+^)^41^, neuron (*SLC17A6*^+^, *STMN2*^+^)^41,42^ and retinal epithelial cell (retinal epi, *RPE65*^+^)^43^. Additionally, we found extraembryonic cells (*VGLL1*^+^, *XAGE2*^+^)^44^ within the teratomas. We then assessed the contributions of teratomas derived from naïve or primed PSCs to both embryonic and extraembryonic lineages (Fig. 3d,e). Naïve PSC-derived teratomas contained more mesoderm (such as aortic endothelium and myeloid cell), endoderm (such as enterocyte and hepatocyte), and extraembryonic lineages. On the contrary, primed PSC-derived teratomas showed enhanced capacity to generate ectoderm cells, such as retinal epithelial cell and radial glia, while their contribution to endodermal and extraembryonic lineages was minimal. The limited contribution of primed H9 ESCs to endoderm is in line with a previous scRNA-seq dataset^11^. In this regard, it should be considered that different PSC lines have different differentiation bias and that in the future it will be interesting to compared teratomas from more cell lines. The cell type annotation was further validated by calculating the correlation of the gene activity score for each cell type in teratomas with gene expression in the corresponding cell types from human fetal tissues aged 72 to 129 days^1^. Most teratoma cell types showed significant correlation with at least one matching cell type in the fetal tissues (Fig. 3f). For instance, the endodermal cells in teratomas exhibited high correlation with human fetal endodermal cell types such as goblet cell, hepatoblast and intestinal epithelial cell, while the extraembryonic cell demonstrated closer correlation with fetal extraembryonic cell types.

**Fig. 4:**
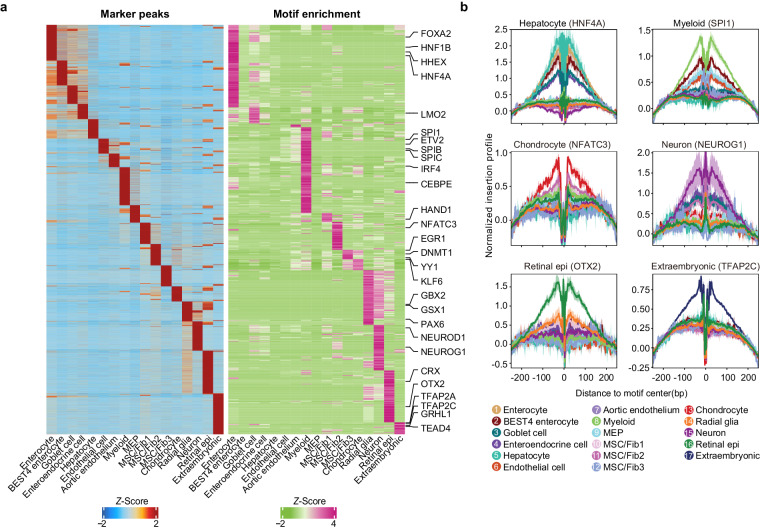
Identification of key regulatory TFs for both embryonic and extraembryonic lineages. (**a**) Heatmap showing the cell type-specific peaks (left panel), and enriched motifs in these differentially accessible regions (right panel). MEP: megakaryocyte-erythroid progenitor, Retinal epi: retinal epithelial cell, MSC/Fib: mesenchymal stem cell/fibroblast. (**b**) Line plot showing the footprint of representative cell-type specific TFs in scATAC-seq data.

To investigate the TFs in cell fate specification, we first conducted peak calling using MACS2^45^ for each cell type to identify cell type-specific chromatin accessible regions. These peaks were then merged to yield a total of 613,760 reproducible peak sets, with 242,353 classified as differentially accessible across the 17 cell types (Fig. 4a). Of these cell type-specific cis-regulatory elements (cCREs), 25.32% were promoters (±3 Kb around TSS) and 74.68% were enhancers. We next applied motif enrichment analysis to the cell type-restricted cCREs to reveal potential regulators of cell fate specification. For instance, analyzing cCREs specific to myeloid cells, we found them enriched for binding sites of the E26-transformation-specific (ETS) transcription factor family, including SPI1. Of note, SPI1 is a crucial regulator of the myeloid lineage^46^. Additionally, cCREs restricted to extraembryonic lineages were enriched for the binding sites of AP-2 family members such as TFAP2C. The latter TF is an important regulator of human extraembryonic lineage specification and maintenance^47,48^. We also identified other well-known cell-type-specific motifs, including HNF4A specific to hepatocyte, NFATC3 specific to chondrocyte, NEUROG1 specific to neurons, and OTX2 motif to retinal epithelial cells^49–52^. Footprinting analysis corroborated the cell type-specific differential motif enrichment (Fig. 4b).

Our study provides a comprehensive dataset of the cis-regulatory logic of cell fate specification during multilineage differentiation in human PSC-derived teratomas. Knowledge resulting from further exploration of this dataset will have broad implications for enhancing our understanding of human embryonic development, disease prevention, and the production of functional cells for therapeutic applications. Systematic experiments involving more PSC lines and other naïve media will be relevant to ensure the applicability and generalizability of the findings.

## Usage Notes

The DNBelab C Series Single-Cell ATAC Library Prep Set (MGI, 1000021878) utilized in this study involved super-loading with beads, enabling the inclusion of multiple beads in each droplet. Before performing downstream analysis, the beads were deconvoluted according to the read mapping loci in each library. The DNBelab C Series scATAC-seq analysis pipeline (https://github.com/MGI-tech-bioinformatics/DNBelab_C_Series_HT_scRNA-analysis-software) is the standard data processing pipeline for scATAC-seq datasets generated by DNBelab C Series Single-Cell ATAC Library Prep Set. The output of the DNBelab C Series scATAC-seq analysis pipeline can be used directly with ArchR package^24^ for downstream analysis.

## Data Availability

Analysis in this study, including data processing and downstream analysis, were conducted according to the standard code of DNBelab C Series scATAC-seq analysis pipeline and ArchR package^24^. The R codes used to analyze the data in this study were available at Figshare^29^ (10.6084/m9.figshare.24547609.v2).
